# Synchronization in epidemic growth and the impossibility of selective containment

**DOI:** 10.1093/imammb/dqab013

**Published:** 2021-10-25

**Authors:** Jan C Budich, Emil J Bergholtz

**Affiliations:** 1 Institute of Theoretical Physics, Technische Universität Dresden and Würzburg-Dresden Cluster of Excellence ct.qmat, 01062 Dresden, Germany; 2 Department of Physics, Stockholm University, AlbaNova University Center, 106 91 Stockholm, Sweden

## Abstract

Containment, aiming to prevent the epidemic stage of community-spreading altogether, and mitigation, aiming to merely ‘flatten the curve’ of a wide-ranged outbreak, constitute two qualitatively different approaches to combating an epidemic through non-pharmaceutical interventions. Here, we study a simple model of epidemic dynamics separating the population into two groups, namely a low-risk group and a high-risk group, for which different strategies are pursued. Due to synchronization effects, we find that maintaining a slower epidemic growth behaviour for the high-risk group is unstable against any finite coupling between the two groups. More precisely, the density of infected individuals in the two groups qualitatively evolves very similarly, apart from a small time delay and an overall scaling factor quantifying the coupling between the groups. Hence, selective containment of the epidemic in a targeted (high-risk) group is practically impossible whenever the surrounding society implements a mitigated community-spreading. We relate our general findings to the ongoing COVID-19 pandemic.

The ongoing COVID-19 pandemic caused by the new Coronavirus SARS-CoV-2 is among the biggest global challenges of our time (WHO, 2020), and its quantitative analysis has thus been an intense focus of recent research (Ferguson *et al.*, 2020; Flaxman *et al.*, 2020; Britton *et al.*, 2020; Salje *et al.*, 2020; Kucharski *et al.*, 2020; nCoV-2019 Data Working Group, 2020; Contreras *et al.*, 2021; Contreras *et al.*, 2021; Sturniolo *et al.*, 2021; Chikina & Pegden, 2020). A repeatedly debated (Kulldorf *et al.*, 2020; Alexanderson *et al.*, 2020) mitigation strategy is based on selectively protecting vulnerable individuals that are at high risk to die or at least develop a severe condition when contracting the virus. Such a strategy, in the following referred to as 2GROUPS, in practice amounts to defining (at least) two groups of individuals, a low-risk group (L) and a high-risk group (H), and then focusing most of the available resources to try and protect the group H from infection, while the larger low-risk group basically does ‘business as usual’, possibly combined with moderate general mitigation measures aimed at ‘flattening the curve’ of infections for group L.

Here, we analyse the expected qualitative outcome of such a 2GROUPS scenario.^1^ Generally speaking, there are two crucial ingredients to a 2GROUPS strategy: first, efficient criteria to identify the high-risk and the low-risk individuals a priori. Regarding this aspect, earlier studies indicated that a large fraction of anticipated severe cases may be concentrated in a relatively small group H, even if only the single criterion of age is used (Ferguson *et al.*, 2020; ourworldindata.org, 2021). Second, the isolation of the high-risk group aimed at strongly containing the prevalence of the disease in group H. In this regard, we find that the selective isolation of the risk group is largely stymied by synchronization effects in the equations governing the pandemic dynamics (see Fig. 1 for an illustration). In particular, we analytically demonstrate within a minimal mathematical model that maintaining a qualitatively slower growth of infections in the high-risk group is unstable against any finite coupling between the two groups, and thus impractical. Instead, the overall exposure of the group H is found to be proportional to that of group L, which may allow for a certain degree of mitigation within H, but largely rules out selective containment within the high-risk group. We note that quantitative numerical simulations of age-targeted mitigation strategies for the specific case of the USA have recently been carried out (Chikina & Pegden, 2020), however without posing the qualitative questions analysed here.

**Fig. 1.**
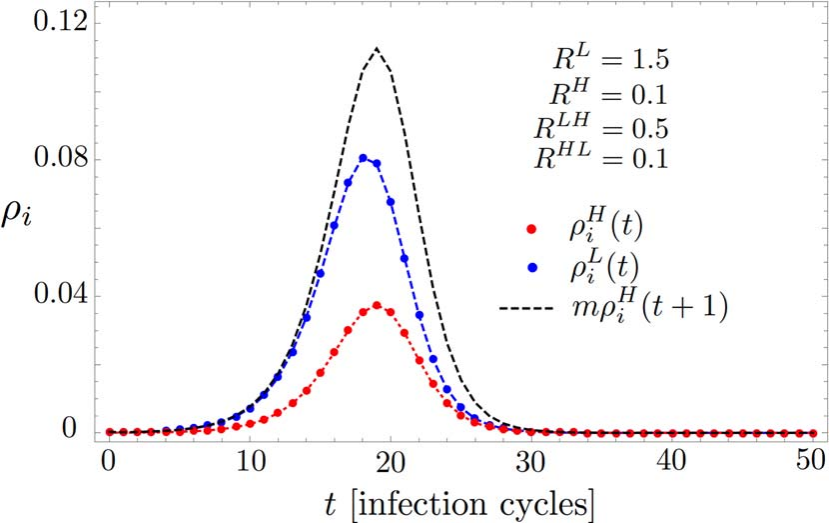
Syncronization of epidemic growth governed by (1). Even if the high-risk group (H) isolates very efficiently, here quantified by a small basic reproduction number $R^{LH}=0.5$, a significant fraction $\rho _i^H$ (red) becomes infected if the low-risk group (L) exhibits a mitigated ‘flatten the curve’ scenario, here at $R^{H}=1.5$, as described by $\rho _i^L$ (blue). We show that this holds for any finite coupling between the groups due to a synchronization effect taking place in exponential growth phase of the epidemic. Specifically, during the synchronization the high and low-risk group infection rates are scaled by a simple constant factor, $m=R^{L}/R^{LH}$ and shifted by one time-step (cf. the black dashed curve). After the synchronization the infection rate in the high-risk group remains significant and bounded from below as $m\rho _i^H(t+1)\geq \rho _i^L(t)$. Initial conditions set to $\rho _i^H(t=0)= \rho _i^L(t=0)=10^{-4}$.

*Minimal mathematical model of 2GROUPS.* We analyse a simple two-component model of SIR type (Kermack & McKendrick, 1927; Murray, 2010; Hethcote, 2006; Muroya *et al.*, 2013; Jia *et al.*, 2011; Kuniya *et al.*, 2016), in which the qualitative synchronization between the epidemic curves of the two groups (cf. Fig. 1) may be readily understood analytically. The corresponding equations for the low-risk group L read as

(1) \begin{eqnarray*} \frac{\varDelta \rho_s^L}{\varDelta t} &=& - R^{HL} \rho_i^H \rho_s^L-R^L \rho_i^L \rho_s^L, \nonumber\\[-1ex] \frac{\varDelta \rho_i^L}{\varDelta t} &=& R^{HL} \rho_s^L \rho_i^H +R^L \rho_i^L \rho_s^L - \rho_i^L, \nonumber\\[-1ex] \frac{\varDelta \rho_r^L}{\varDelta t} &=& \rho_i^L, \end{eqnarray*}

and for the high-risk group H, we have

(2) \begin{eqnarray*} \frac{\varDelta \rho_s^H}{\varDelta t} &=& - R^{LH} \rho_i^L \rho_s^H-R^H \rho_i^H \rho_s^H, \nonumber\\[-1ex] \frac{\varDelta \rho_i^H}{\varDelta t} &=& R^{LH} \rho_s^H \rho_i^L +R^H \rho_i^H \rho_s^H - \rho_i^H, \nonumber\\[-1ex] \frac{\varDelta \rho_r^H}{\varDelta t} &=& \rho_i^H. \end{eqnarray*}

Here, $\rho _\mu ^\alpha , ~\mu = s, i, r,~\alpha = L,H$ stands for the density (fraction) of individuals within group $\alpha $ that are susceptible ($\mu = s$), infectious ($\mu = i$) and recovered ($\mu = r$), respectively. Explicitly, $\rho _\mu ^\alpha = N_\mu ^\alpha /N^\alpha $ relates the densities to the corresponding absolute numbers. Note that the constraint $\varDelta \rho _s^\alpha +\varDelta \rho _i^\alpha +\varDelta \rho _r^\alpha =0$, consistent with $\rho _s^\alpha +\rho _i^\alpha +\rho _r^\alpha =1$ is automatically fulfilled, reflecting a static total population $N^\alpha $ within each group. The time-step or infection cycle of the discretized dynamics is denoted by $\varDelta t$ and for SARS-CoV-2 roughly amounts to 5 days in real time (Nishiura *et al.*, 2020). In the following, in particular in all plots, time is measured in units of $\varDelta t$. For simplicity, we have also put the recovery rate to one in these units which is not influencing the qualitative conclusions of the present work. The parameter $R^L$ is the reproduction rate within group L, and the inter-group coupling $R^{HL}$ quantifies the transfer of infections from group H to group L. The corresponding equations for the high-risk group H are identical to (1) upon exchanging $H$ and $L$ in all instances, resulting in (2). The resulting parameters $R^{LH}$ and $R^H$ then denote the transfer of infections from L to H, and the effective reproduction rate within H, respectively. The goal of 2GROUPs is of course to keep $R^{LH}$ as low as possible, but it will still be non-zero in any realistic implementation.

It is important to notice that the reproduction numbers, in the mean field picture of the SIR modelling (and assuming a homogeneous contact structure), scale with the relative size of the groups: $R^L$ and $R^{LH}$ are proportional the fraction of the population assigned to the low-risk group while $R^H$ and $R^{HL}$ are proportional with the population fraction in the high-risk group. In a realistic scenario with the low-risk group being about five times as large as the high risk group this directly leads to a factor of five between $R^{LH}$ as compared with $R^H$ and $R^{HL}$, as reflected in the parameters used for the simulations shown in Fig. 1. Moreover, in our example in Fig. 1, we use $R^{LH}=0.5$ that corresponds to an additional three-fold reduction of contacts involving individuals from group H, compared with the already mitigated values (for SARS-CoV-2) within group L of $R^L=1.5$. This, for the high-risk group, amounts to a rather strict isolation at a level of hard lockdowns in Europe during spring 2020, and even similar to the level reached during the lockdown in Wuhan (Rahman *et al.*, 2020).

To gain analytical insight, and motivated by the aforementioned scaling of parameters with relative group size, within a first approximation we neglect both $R^H$ and $R^{HL}$, while keeping $R^{LH}$ and $R^{L}$ finite. This leads to a uni-directional decoupling of the two groups (note that H no longer appears in (1) for the group L), which allows us to get an intuitive feeling for the synchronization of the two groups (Fig. 2(a)). Corrections arising from restoring the neglected couplings are discussed further below. Roughly speaking, this only adds additional channels of infection compared with the simplifying approximation and at least does not make the situation more favourable for the H group. Within our approximation, we may first solve (1) for group L and then plug the time-dependent solution $\rho _i^L(t)$ into the corresponding equations (2) for $H$. Putting $\varDelta t=1$, i.e. measuring time in units of $\varDelta t$, we thus derive

(3) \begin{align*}& \rho_{i}^{H}(t+1) = R^{LH} \rho_{i}^{L}(t)\rho_{s}^{H} (t) = R^{LH} \frac{\rho_{i}^{L}(t)}{\rho_{i}^{H}(t)} \rho_{i}^{H}(t)\rho_{s}^{H}(t)\nonumber\\ &= \tilde R^{H}(t) \rho_{i}^{H}(t) \rho_{s}^{H}(t), \end{align*}

**Fig. 2.**
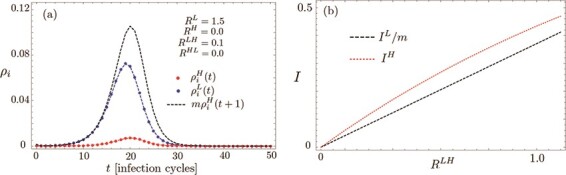
Instability at weak inter-group coupling (a) and total infection during the entire epidemic (b), illustrating (6) for various inter-group coupling values $R^{LH}$ (evaluated at $t=100$, $R^{L}=1.5$, $R^{HL}=R^H=0$ for the initial condition $\rho _i^H(t=0)=\rho _i^L(t=0)=10^{-4}$).

where we have defined $\tilde R^H(t) = R^{LH} \frac {\rho _i^L}{\rho _i^H}$ as an effective time-dependent reproduction rate for group $H$. Equation (3) formally resembles a time-step in a simple epidemic dynamics of a single group but with $R^H$ replaced by the time-dependent $\tilde R^H(t)$. Importantly, since $\tilde R^H(t)$ is proportional to the quotient $\frac {\rho _i^L}{\rho _i^H}$, a much lower density of infected in the H group (the main goal of 2GROUPS) *increases* the epidemic growth within H—a very undesirable but unavoidable effect of the non-linear coupling $R^{LH}$. During the initial exponential growth of infections in L, a stable situation characterized by a time-independent $\tilde R^H$ only occurs if the condition

(4) \begin{align*}& \frac{R^{LH}}{R^L}=\frac{\rho_i^H}{\rho_i^L} \end{align*}

is satisfied. Then, the dynamics of $\rho _i^H$ simply follows the dynamics of $\rho _i^L$ with a delay of a single time step and an overall reduction in amplitude, effectively dividing it by

(5) \begin{align*}& m = \frac{R^{L}}{R^{LH}} \ge 1, \end{align*}

i.e. $m\rho _i^H(t+1)\sim \rho _i^L(t)$ (cf. (4)). Close to the peak and during the downward slope of the dynamics, the delay causes $\rho _i^H$ to grow even slightly above $\rho _i^L/m$ (see Fig. 1). Hence, a simple estimate for the total number of infected $I^H$ within the high-risk group is given by

(6) \begin{align*}& I^H \ge I^L/m, \end{align*}

where $I^L$ denotes the overall total number of infected within the low-risk group during the epidemic (see Fig. 2(b)). These general results are illustrated in Figs 1 and 2(a). In fact, a stronger bound holds throughout the relevant parts of the epidemic, namely that $m\rho _i^H(t+1)\geq \rho _i^L(t)$ (cf. the black dashed and blue solid curves in Fig. 1). We stress that Fig. 1 shows data on the full model (1) with finite parameter values $R^H=0.1$ and $R^{HL} = 0.1$, and the good agreement with our analytical predictions based on neglecting those couplings thus corroborates the robustness of our analytical picture over a wider parameter range.

More generally, we find that an undesirable outbreak within the high-risk group that, apart from a delay by one time step, is qualitatively similar to the behaviour of the low-risk group occurs as soon as $R^{LH}$ is non-zero, even for very small values (see Fig. 2(a)). Furthermore, increasing $R^H$ and $R^{HL}$ is never found to reduce $\rho _i^H(t)$ (or $I^H$ for that matter). Hence, our analytical picture for $R^H=R^{HL}=0$ may be seen as an optimistic lower bound for the infections within the high-risk group.

## Concluding remarks.

In this work, we have shown that the efficiency of a stratified epidemic strategy dividing the population into a low- and a high-risk group is drastically limited by synchronization effects occurring for any finite coupling between the groups. Specifically, the most optimistic hope to maintain a significantly slower effective reproduction rate for the high-risk group as compared with the low-risk group is largely ruled out. We have explicitly demonstrated this analytically in SIR-based 2GROUP models that give a coarse grained mean-field picture, noting that SIR models are microscopically more accurate for the spread of, e.g. influenza viruses than for SARS-CoV-2, as clusters and superspreading events play an important role for the latter. Nevertheless, some authors have attempted to derive quantitative predictions using similar multi-group SIR modelling, including estimating herd-immunity thresholds (Britton *et al.*, 2020), and detailed implications of age-targeted mitigation strategies (Chikina & Pegden, 2020). Here instead, noting that quantitative predictions may be sensitive to fine-tuning, we focus on the robust qualitative behaviour based on analytical insights rather than on large scale computing. Indeed, we expect that certain quantitative aspects such as the delay in infections between the two groups may differ significantly due to finer structures of a real society not captured by our simple modelling.^2^ Finally, we note that our model assumes perfect immunity upon recovery. That may be a reasonable approximation during a specific wave of the epidemic, and deviations from this assumption can for sure only make the situation worse for both groups.

Despite our minimal modelling, we expect the qualitative effects revealed in our present work, in particular the impossibility of sustaining a selective containment for a risk-group only, to be clearly visible also in more microscopic modelling scenarios. Along these lines, we hope that our findings stimulate future efforts to analyse more detailed COVID-19 specific models from a viewpoint of epidemic curves corresponding to different groups, including effects such as partial immunity, time-dependent strategies, imperfect vaccinations and relaxing NPIs. At the same time, we stress the importance and value of simple analytically amenable models for the basic phenomenological understanding, whereas the complexity of detailed simulations often tend to obfuscate key insight. In many other fields of science, ranging from physics to economics, such simple models have indeed proven central for progress.

As a matter of fact, a sort of 2GROUPs strategy has been applied in Sweden for the first 6–7 months of the COVID-19 pandemic. While this has certainly contributed to keeping the fatalities lower than in an entirely unmitigated scenario, the comparably high fatality rates in Sweden may serve as a practical example of how hard it is to selectively protect high-risk groups (Brandén *et al.*, 2020; Roxby & Gure, 2020). While insufficient security measures naturally play a major role here, we note that the outcome is in agreement with our general analysis: the substantial impact on the high-risk group has been reflecting the high spread in society at large. This stands in contrast to the neighbouring countries that have applied a containment strategy directed to the society at large. Even without similarly stringent restrictions for the risk groups, this has resulted in a per capita fatality rate roughly an order of magnitude lower than in Sweden.

This indicates that synchronization phenomena as the ones revealed in our present study might be more universal for epidemic dynamics, as long as infections occur between individuals (as opposed to disease spreading via agents such as mosquitos). Consistent with this picture, a recent systematic study shows a very strong correlation across many countries between the number of fatalities in care homes and in the population as a whole Comas-Herrera *et al.*, (2021).

## Footnotes

1 We do not attempt a detailed modeling with many parameters specific to a given country, but rather aim at distilling robust qualitative properties of the considered scenarios that do not depend on any fine-tuning. Quantitative predictions will be about orders of magnitude rather than changes of a few percent.

2 An example of this is that the infection may be introduced to society in a specific subpopulation of the low-risk groups, such as rich and healthy tourists that go on skiing vacations, while it takes a few time steps (generations of transmission) until it has reached e.g. the usually poorer care home workers that are in direct contact with the high-risk group.

## References

[ref1] Alexanderson, K. *et al.* (2020) The John Snow Memorandum. https://www.johnsnowmemo.com.

[ref2] Brandén, M. *et al.* (2020) Residential context and COVID-19 mortality among adults aged 70 years and older in Stockholm: a population-based, observational study using individual-level data. Lancet Healthy Longev., 2020, S2666–S7568. 10.1016/S2666-7568(20)30016-7 PMC7832817 33521770

[ref3] Britton, T., Ball, F. & Trapman, P. (2020) A mathematical model reveals the influence of population heterogeneity on herd immunity to SARS-CoV-2. Science, 369, 846–849. 32576668 10.1126/science.abc6810 PMC7331793

[ref4] Chikina, M. & Pegden, W. (2020) Modeling strict age-targeted mitigation strategies for COVID-19. PLoS ONE, 15, e0236237. 32706809 10.1371/journal.pone.0236237 PMC7380601

[ref5] Comas-Herrera *et al.* (2021) Mortality associated with COVID-19 in care homes: international evidence. Article in LTCcovid.org, International Long-Term Care Policy Network, CPEC-LSE.

[ref6] Contreras, S. *et al.* (2020) Low case numbers enable long-term stable pandemic control without lockdowns. medRxiv: https://doi.org/10.1101/2020.12.10.20247023. 10.1126/sciadv.abg2243 PMC8500516 34623913

[ref7] Contreras, S. *et al.* (2021) The challenges of containing SARS-CoV-2 via test-trace-and-isolate. Nat. Commun., 12, 378. 33452267 10.1038/s41467-020-20699-8 PMC7810722

[ref9] ourworldindata.org . (2021) Case fatality rate of COVID-19 by age. https://ourworldindata.org/mortality-risk-covid#case-fatality-rate-of-covid-19-by-age.

[ref11] Ferguson, N. M. *et al.* (2020) Impact of non-pharmaceutical interventions (NPIs) to reduce COVID-19 mortality and healthcare demand. https://doi.org/10.25561/77482. 10.1007/s11538-020-00726-x PMC7140590 32270376

[ref12] Flaxman, S. *et al.* (2020) Estimating the number of infections and the impact of non-pharmaceutical interventions on COVID-19 in 11 European countries (Imperial College London, 2020). https://doi.org/10.25561/77731.

[ref13] Hethcote, H. W. (2006) The mathematics of infectious diseases. SIAM Rev., 42, 599.

[ref14] Jia, C., Jiang, D. & Shi, N. (2011) Multigroup SIR epidemic model with stochastic perturbation. Phys. A, 390, 1747.

[ref15] Kermack, W. O. & McKendrick, A. G. (1927) A contribution to the mathematical theory of epidemics. Proc. Roy. Soc. A, 115, 700–721.

[ref16] Kucharski, A. J. *et al.* (2020) Early dynamics of transmission and control of COVID-19: a mathematical modelling study. Lancet Infect. Dis., 20, 553–558. 32171059 10.1016/S1473-3099(20)30144-4 PMC7158569

[ref17] Kulldorf, M. *et al.* (2020) The Great Barrington Declaration. https://gbdeclaration.org.

[ref18] Kuniya, T., Wang, J. & Inaba, H. (2016) A multi-group SIR epidemic model with age structure. Discrete Contin. Dyn. Syst. Ser. B, 21, 10.

[ref19] Muroya, Y., Enatsu, Y. & Kuniya, T. (2013) Global stability of extended multi-group sir epidemic models with patches through migration and cross patch infection. Acta Math. Sci. (Chinese), 33, 341.

[ref20] Murray, J. (2010) Mathematical Biology. New York, NY, USA: Springer.

[ref21] nCoV-2019 Data Working Group (2020) Epidemiological data from the nCoV-2019 outbreak: early descriptions from publicly available data. http://virological.org/t/epidemiological-data-from-the-ncov-2019-outbreak-early-descriptions-from-publicly-available-data/337.

[ref22] Nishiura, H., Linton, N. M. & Akhmetzhanov, A. R. (2020) Serial interval for novel coronavirus (COVID-19) infections. Int. J. Infect. Dis., 93, 284–286. 32145466 10.1016/j.ijid.2020.02.060 PMC7128842

[ref23] Rahman, B., Sadraddin, E. & Porreca, A. (2020) The basic reproduction number of SARS-CoV-2 in Wuhan is about to die out, how about the rest of the world? Rev. Med. Virol., 30, e2111. 32431085 10.1002/rmv.2111 PMC7267092

[ref24] Roxby, A. C. & Gure, T. R. (2020) Lessons from Sweden: where can older adults shelter from COVID-19? Lancet Healthy Longev., 1, E53–E54. 34173616 10.1016/S2666-7568(20)30035-0 PMC7598753

[ref25] Salje, H. *et al.* (2020) Estimating the burden of SARS-CoV-2 in France. Science, 369, 208–211. 32404476 10.1126/science.abc3517 PMC7223792

[ref26] Sturniolo, S. *et al.* (2021) Testing, tracing and isolation in compartmental models. PLoS Comput. Biol., 17, e1008633. 33661888 10.1371/journal.pcbi.1008633 PMC7932151

[ref27] WHO (2020) Coronavirus Disease 2019 (COVID-19): Situation Report - 72. Geneva: World Health Organization.

